# Monoclonal Gammopathy of Undetermined Significance with Amyloid Deposition in the Lung and Non-Amyloid Eosinophilic Deposition in the Brain: A Case Report

**DOI:** 10.1155/2010/406102

**Published:** 2010-03-14

**Authors:** Francois Abi-Fadel, Nisarg R. Desai, Gita Vatandoust, Rabih Said, Aaron Gottesman, Terenig Terjanian

**Affiliations:** ^1^Internal Medicine Department, Staten Island University Hospital, 475 Seaview Avenue, Staten Island, NY 10305, USA; ^2^Hematology & Oncology Department, Staten Island University Hospital, 475 Seaview Avenue, Staten Island, NY 10305, USA; ^3^Director Hospitalist Services, Staten Island University Hospital, 475 Seaview Avenue, Staten Island, NY 10305, USA

## Abstract

*Background*. Monoclonal gammopathy of undetermined significance (MGUS) is rarely complicated by amyloidosis. *Case*. A 66-year-old white male presented to the emergency room (ER) after an unwitnessed fall and change in mental status. Patient was awake and alert but not oriented. There was no focal deficit on neurological exam. Past medical history (PMH) included hypertension, hypercholesterolemia, aortic valve replacement (nonmetallic), incomplete heart block controlled by a pacemaker and IgG- IgA type Monoclonal Gammopathy of Undetermined Significance. The MGUS was diagnosed 9 months ago on serum protein electrophoresis (SPEP) as patient was referred to the outpatient clinic for hyperglobulinemia on routine blood work. In ER, a head-computed tomography (CT) revealed multiple parenchymal hemorrhagic lesions suspicious for metastases. A CT chest, abdomen and pelvis revealed numerous ground-glass and solid nodules in the lungs. Lower extremity duplex and transesophageal echocardiogram were negative. Serial blood cultures and serologies for cryptococcus and histoplasmosis, antineutrophil cytoplasmic antibody (ANCA), antinuclear antibody (ANA), rheumatoid factor (RF), cryoglobulin, and antiglomerular basement membrane (anti-GBM) antibodies were all negative. CT guided lung biopsy was positive for Thioflavin T amyloid deposits. Brain biopsy was positive for eosinophilic material (similar to the lungs) but negative for Thioflavin T stain. The patient's clinical status continued to deteriorate with cold cyanotic fingers developing on day 12 and a health care acquired pneumonia, respiratory failure, and fungemia on day 18. On day 29, family withdrew life support and denied any autopsies. *Conclusion*. Described is an atypical course of MGUS complicated by amyloidosis of the lung and nonamyloid eosinophilic deposition in the brain. As MGUS might be complicated by diseases such as amyloidosis and multiple myeloma, a scheduled follow-up of these patients is always necessary. Further research is needed in order to better define the optimal treatment and management strategies of MGUS and its complications.

## 1. Introduction

B cell dyscrasias are characterized by a clonal proliferation of B cells (benign or malignant). The spectrum of B cell dyscrasia is broad and includes the monoclonal gammopathy of undetermined significance (MGUS) [1]. MGUS is found in more than 1% of persons who are 50 years or older and is defined by the presence of a serum monoclonal protein concentration less than 3 g/dL, with absence of lytic bone lesions, of M proteins in urine, of hypercalcemia and of renal failure [1–3]. Amyloidosis is a rare complication of MGUS which is secondary to excessive production of a monoclonal immunoglobulin (Ig) and deposition of the single Ig isotype or subunits in various tissues. Aggregomas, on the other hand, are tumoral non-amyloidotic monoclonal immunoglobulin light chain deposits [4]. In this paper, we describe an unusual presentation of MGUS combining amyloid deposition in the lung and non-amyloid eosinophilic deposition in the Brain.

## 2. Case

A-66-year-old male, who was exsmoker, presented to the ER for a change in mental status. As per the family, the patient who had a normal mental status in the past was noticed to be progressively worsening for the last couple of months, with disorientation mainly found over phone discussions. At the time of presentation he was awake and alert, but disoriented to time and place. He denied any pain and was unable to provide any history. Physical exam was normal except for the presence of facial ecchymosis. The neurological part showed a normal motor, sensory, and cranial nerve exam. Also, his gait was stable. 

Past medical history was significant for hypertension, hypercholesterolemia, aortic valve replacement (nonmetallic), atrial fibrillation (on warfarin), incomplete heart block, pacemaker, and IgG- IgA, kappa type Monoclonal Gammopathy of Undetermined Significance (MGUS). Family history was not relevant.

MGUS was diagnosed 9 months ago on serum protein electrophoresis (SPEP) as patient was referred to our institution's center for cancer and blood related diseases for hyperglobulinemia on routine blood work. At that time the patient underwent extensive work up to rule out any plasma cell dyscrasias. The Urine protein electrophoresis (UPEP) was negative for M spike. Immunohistochemistry and flow cytometry from peripheral smear as well as from bone marrow biopsy were negative for a clonal plasma cell or malignant B-cell. Chromosomal analysis and cytogenetics were also normal. 

In the ER, a head CT scan revealed multiple parenchymal hemorrhagic lesions suspicious for metastases mainly in the frontal part bilaterally and in the left parieto-temporal region, with edema and compression mainly on the left lateral ventricle. Also, an interventricular lesion at the foramen of Monro causing mild hydrocephalus was seen. CT chest, abdomen and pelvis was done to find a possible primary malignancy and revealed numerous ground-glass and solid nodules in the lungs. Warfarin was discontinued and the international normalized ratio (INR) of 2.2 was reversed with fresh frozen plasma. Patient was started on steroids and phenytoin. Lower extremity duplex was negative.

**Figure 1:**
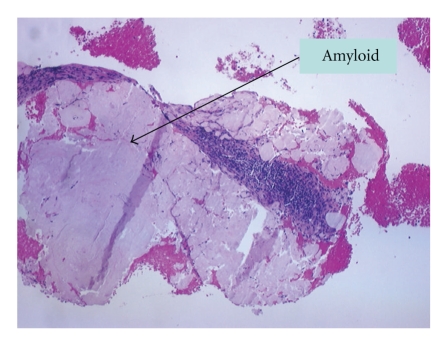
CT guided lung biopsy showed H&E (hematoxylin and eosin) and PAS (periodic acid-schiff) positive amorphous eosinophilic material suspicious for amyloid. Initial staining with congo red was negative. However, it stained positively with Thioflavin T stain and had weak green birefringence under polarized light.

**Figure 2:**
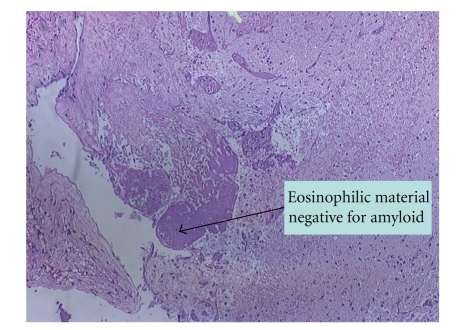
Brain biopsy was positive for eosinophilic material (similar to the lungs) but negative for congo red and thioflavin T stain.

The clinical status continued to deteriorate with cold, cyanotic fingers developing on day twelve of hospitalization. At this point he was seen by a vascular surgeon in order to rule out arterial emboli. A transesophageal echocardiogram was done for possible vegetations causing infectious emboli. All results were negative, and no infiltrative cardiomyopathy was found. Bronchoscopy and broncho-alveolar lavage with cultures (including nocardia) was also negative. CT guided lung biopsy showed H&E (hematoxylin and eosin) and PAS (periodic acid-schiff) positive amorphous eosinophilic material suspicious for amyloid (Figure 1). Initial staining with congo red was negative. However, it stained positively with Thioflavin T stain and had weak green birefringence under polarized light. Immunostains for kappa and lambda chains showed stronger staining for kappa subtype. Brain biopsy was positive for eosinophilic material (similar to the lungs) but negative for congo red and thioflavin T stain (Figure 2). Later, the patient had a respiratory failure secondary to health care acquired pneumonia (HCAP) and was intubated. Serial blood cultures and serologies for Cryptococcus and histoplasmosis, serology for antineutrophil cytoplasmic antibody (ANCA), antinuclear antibody (ANA), rheumatoid factor (RF), cryoglobulin, and Antiglomerular basement membrane antibodies (antiGBM) were all negative. Only seen was a positive blood culture for candida on day eighteen. 

Patient was initially treated with ampicillin and gentamycin for possible endocarditis. Later, he was switched to vancomycin, meropenem, and ciprofloxacin for possible health care acquired pneumonia. Also, he was started on amphotericin B after blood cultures revealed candida. Whether a more diffuse disease such as systemic amyloidosis is present was not confirmed, as the family refused any further diagnostic procedures that are not relevant to the acute care of the patient in a context of a deteriorating clinical picture. Despite all antifungals and supportive measures, he failed weaning trials and remained on ventilator support. A tracheostomy was suggested. However, after extensive discussions, the family refused. On day 29, health care proxy decided to withdraw all life support measures based on the patient's previous wishes who did not want to be kept alive on a ventilator. Finally, the patient had a terminal wean after these events, and the family refused any autopsies.

## 3. Discussion

As we discussed above, MGUS is found in around 1% of persons who are 50 years or older and is defined by the presence of a serum monoclonal protein concentration less than 3 g/dL. MGUS of IgM class can progress to lymphoma and Waldenstrom macroglobulinemia; whereas IgA and IgG can progress to primary amyloidosis or multiple myeloma. B cell lymphoproliferative disorders can cause AL (light chain) amyloidosis, which is very rarely reported in MGUS [1, 4]. Depending on the type of antibodies secreted, immunoglobulin deposition diseases can be divided into light chain amyloidosis (AL), heavy chain amyloidosis (AH), non-amyloid forms of light chain deposition diseases (LCDD), and heavy chain deposition diseases (HCDD). Coexistence of the amyloid and non-amyloid deposits is very rare, and was first described by Gallo et al. as “monoclonal immunoglobulin deposition diseases” [5]. 

Biclonal gammopathy was described in less than 3 percent of the Mayo clinic MGUS series [3]. The clinical course and behavior were not significantly different. Our patient had high levels of IgG 3730 mg/dL (range: 751–1560 mg/dL) and IgA 619 (range: 82–453 mg/dL). Immunofixation was positive for kappa chains which was consistent with the amyloid deposits in the lungs. These were highly positive for the kappa immunostaining. Therefore, we can suggest that one of the contributing causes for the respiratory failure in our case might have been the amyloidosis itself. 

Also, found in our patient was multiple organizing hemorrhagic brain lesions suspicious for metastasis. However, no primary tumor was found and there was no evidence of lymphoproliferative diseases in the brain or lung biopsies. Hemorrhage in patients with MGUS can be secondary to amyloidosis or cryoglobulinemia. However, finding of eosinophilic non-amyloid material possibly suggests “Aggregoma” which is a very rare entity, only described once previously in the literature, as per our knowledge [4]. Amyloid depositions seen in plasma cell dyscrasias can lead to amyloid angiopathy and secondary hemorrhagic lesions [6, 7]. Light chain deposition diseases might mimic amyloidosis, leading also to bleeding susceptibilities [6, 7]. Our patient might have light chain deposition disease in the brain that did not lead to amyloidosis itself. However, this disease might have been the cause of significant parenchymal hemorrhages. 

Further workup in order to define the exact nature of these depositions, like immunohistochemistries, ultra-structural analysis, and mass spectrometry, was not done, mainly due to technical and technological limitations in our institution. Also, we cannot rule out a possible systemic amyloidosis as no further diagnostic testing such as a fat pad biopsy was done. The family refused any workup that is not relevant to the acute care of the patient who was in a critical condition. Also, the final diagnosis was not ascertained as the autopsy was refused.

Although there were some indicators predicting the risk of progression to Multiple Myeloma (serum protein M ≥ 1.5, non-IgG MGUS), till now, there is no known indicators predicting the odds of amyloidosis formation. Periodic monitoring of MGUS is advised, but there is currently no recommended optimal intervals. Many experts recommend routine follow-up between 6 and 12 months. This is based mainly on the potential risk for multiple myeloma progression. 

## 4. Conclusion

This case describes two organ specific presentations related to the MGUS disease: amyloid deposition in the lung and non-amyloid eosinophilic deposition in the brain. The possibility of a presence of systemic amyloidosis and the final confirmation of the nature of the non-amyloid depositions in our case were not ascertained, mainly due to the refusal by the family for any autopsies or any further diagnostic procedures, but also view the lack of technological availabilities, mainly staining subtypes in our institution.

Knowing that the course of MGUS can be potentially complicated by amyloidosis, peripheral neuropathies, and multiple myeloma transformations, a scheduled follow-up of these patients is always necessary. Further research is needed in order to establish the optimal treatment guidelines for the complications and diseases related to MGUS.

##  Consent

Written informed consent was obtained from the patient's next of kin for publication of this case report and accompanying images. A copy of the written consent is available for review by the Editor-in-Chief of this journal.

##  Competing Interests

The authors declare that they have no competing interests.

##  Author's Contributions

N. Desai, G. Vatandoust, R. Said, T. Terjanian, and F. Abi-Fadel were involved in the care of the patient, and in establishing the diagnosis. N. Desai and F. Abi-Fadel reviewed the literature, and collaborated in writing the paper. A. Gottesman and T. Terjanian provided the final review of the paper. All authors read and approved the final paper.
